# Management of staghorn renal stones

**DOI:** 10.1080/0886022X.2018.1459306

**Published:** 2018-04-16

**Authors:** Akif Diri, Banu Diri

**Affiliations:** a Faculty of Medicine, Aksaray University, Aksaray, Turkey;; b Department of Urology and Nephrology, Aksaray University, Aksaray, Turkey

**Keywords:** Kidney, percutaneous nephrolithotomy, staghorn stone, struvite, management

## Abstract

Staghorn stones are large branching stones that fill part of all of the renal pelvis and renal calyces and they can be complete or partial depending on the level of occupancy of the collecting system. Although kidney stones are commoner in men, staghorn stones are less often reported in men compared to women and they are usually unilateral. Due to the significant morbidity and potential mortality attributed to staghorn stones, prompt assessment and treatment is mandatory. Conversely, conservative treatment has been shown to carry a mortality rate of 28% in 10-year period and 36% risk of developing significant renal impairment. Staghorn stones are, therefore, significant disease entity that should be managed aggressively and effectively. Generally, the gold standard treatment for staghorn stones is surgical with a view to achieve stone-free collecting system and preserve renal function. Percutaneous nephrolithotomy should be the recommended first-line treatment for staghorn stones. Other non-surgical options are usually considered in combination with surgery or as monotherapy only if patients are surgically unfit. The decision for optimal treatment of staghorn stones should be individualized according to the circumstances of the patient involved and in order to do so, a closer look at the advantages and disadvantages of each option is necessary.

## Introduction

Staghorn stones are large branching stones that fill part of all of the renal pelvis and renal calyces and they can be complete or partial depending on the level of occupancy of the collecting system [1]. Although, the term ‘staghorn’ provides description of stone configuration, it lacks specific volume criteria and information about stone composition [2]. Previously, it was widely accepted that staghorn stones formed 10–20% of the entire urinary stones; however, this figure is currently reduced to 4% in developed countries due to early and effective management of renal stones [3].

Although kidney stones are commoner in men, staghorn stones are less often reported in men compared to women and they are usually unilateral [4–8]. Staghorn stones are infection stones in 49–68% of cases and, therefore, the term staghorn traditionally referred to struvite stone [9,10]. Struvite stone, first described by a Swedish geologist named Ulex in 1845, is composed of magnesium, ammonium, and phosphate and it is closely related to urinary tract infection caused by urease-producing organisms, namely Proteus, Klebsiella, Pseudomonas, and Staphylococcus bacteria [1,11].

Due to the significant morbidity and potential mortality attributed to staghorn stones, prompt assessment and treatment is mandatory. Conversely, conservative treatment has been shown to carry a mortality rate of 28% in 10-year period and 36% risk of developing significant renal impairment [12,13]. Staghorn stones are, therefore, significant disease entity that should be managed aggressively and effectively. We herein provide an overview on the pathophysiology and evidence-based treatment options of unilateral staghorn calculus in adult patients.

## Main body

### Mechanism of struvite stone formation

The formation of struvite kidney stone requires an increase in urinary ammonia production accompanied by an increase in urinary pH, reducing phosphate solubility [14]. For this to occur, the presence of urease-producing bacteria is necessary [15]. Organisms producing urease enzyme splits urinary urea into ammonia which, in turns, hydrolyzes to bicarbonate and ammonium. These will then forms magnesium ammonium phosphate and carbonate apatite upon binding to cations. Bacteria also metabolize the citrate in urine and stop its protective binding to calcium and phosphate.

Crystallization, both inside and outside the bacteria, is facilitated by the formation of struvite–apatite dust. Intra-bacterial crystallization causes bacteriolysis and microlith formation which acts like a nidus for stone formation. Peri-bacterial crystals, on the other hand, form a cover that encloses the bacteria and allows it to act as a source of recurrent infections [15].

### Factors predisposing to unilateral staghorn stone formation

Apart from urinary tract infections by urease-producing bacteria explained above, there are other factors to blame for staghorn stone formation. These include, urinary tract obstruction or anatomical abnormalities, long-term use of indwelling urethral catheter, previous urinary diversion surgery and, lastly, neurogenic bladder pathology [16].

### Changing composition of staghorn stone – recent views

Despite the historical fact that large proportion of staghorn stones are made of struvite, recent reports revealed an increasing number of calcium phosphate staghorn stones, supporting the link between staghorn and metabolic stones [14,17]. Although, to date, there are no convincing explanations for the shift in staghorn stones composition, but it is thought to be secondary to geographical, dietary, and lifestyle changes [18].

Also, staghorn stones sometimes present as mixed stones – composed of calcium carbonate apatite and struvite. This could possibly be explained by the primary tendency to form calcium oxalate stones which subsequently harbor bacteria triggering the cascade of events leading to secondary struvite deposition [19]. Accordingly, changing composition of staghorn stones clearly suggests that metabolic evaluation of such patients is necessary as metabolic stones are easier to prevent than struvite ones [10].

### Treatment options for unilateral staghorn stone

Some clinicians, until the 1970s, believed that staghorn stones should be left alone – no active treatment [20]. However, improved knowledge of the natural history of staghorn stones has led to significant change in its management objectives. Currently, it is agreed that untreated staghorn stones could lead to significant morbidity and mortality. Generally, the gold standard treatment for staghorn stones is surgical with a view to achieve stone-free collecting system and preserve renal function. Other non-surgical options are usually considered in combination with surgery or as monotherapy only if patients are surgically unfit.

However in a recent study, Deutsch and Subramonian [21] showed that conservative management of staghorn calculi, in the context of patients who are unfit for surgery or who decline intervention, can be a suitable option. They also concluded that conservative management of staghorn calculi is not as unsafe as previously thought.

The decision for optimal treatment of staghorn stones should be individualized according to the circumstances of the patient involved and in order to do so, a closer look at the advantages and disadvantages of each option is necessary [22].

#### Percutaneous nephrolithotomy (PCNL)

PCNL was first introduced in 1970s to treat small renal stones. Its subsequent role in the management of staghorn stones was facilitated by the availability of ultrasonic and electrohydraulic lithotripters [23].

In a large series, PCNL for staghorn stones revealed complete stone clearance rates of 98.5% and 71% for partial and complete staghorn stones, respectively. The overall complication rate in this study was as low as 4% [24]. Although very popular, surprisingly there are only two randomized controlled trials (RCT) assessing the therapeutic value of PCNL. The first RCT was published in 2005 and concluded superiority of PCNL over extracorporeal shock wave lithotripsy (ESWL) in the management of staghorn stones [25]. Therefore, PCNL is recommended as the first-line treatment for struvite staghorn stones [2,22,26].

The second RCT examined the role of PCNL in treating staghorn stones compared to open surgery and showed comparable stone clearance, less bleeding, shorter operative time, less operative complications, and shorter hospital stay in PCNL arm [27]. In 2002, a case series suggested the use of holmium:yttrium-aluminum-garnet (YAG) laser and flexible nephroscope via single renal access [28]. On the other hand, owing to the complexity and branching characteristics of staghorn stone, multiple-access PCNL was popularized by other researchers [29].

Last studies also demonstrated that mini-PCNL is a feasible option for treating patients with complex renal stones, including staghorn stones [30,31]. Mini-PCNL can be used either as one-stage or two-stage procedure in the management of staghorn kidney stones. In a recent report, Zhao et al. [30] suggested that ‘2-stage’ treatment plan may be more prudent. Regardless of the initial treatment plan, a significant proportion of staghorn stones required multiple percutaneous nephrostomy tracts and more than 1 stage of percutaneous procedure to achieve satisfactory result [30].

Ultrasonic and pneumatic devices are the most common used lithotripters during PCNL. The high-power holmium laser lithotripsy (HP-HLL) is now also used for disintegration of stones. In a recent study, El-Nahas et al. [32] compared HP-HLL and ultrasonic lithotripsy (US-L) for disintegration of staghorn stones during PCNL. HP-HLL showed comparable safety and efficacy with a lower hemoglobin deficit but longer operative time when compared with US-L. In the conclusion, authors recommended HP-HLL as a viable treatment option for safe and effective disintegration of staghorn stones during PCNL.

#### Ureterorenoscopy (URS)

Surprisingly, to date, the use of ureteroscopy for staghorn stones as stand-alone treatment has not been published in the literature. Despite that, recent technological advances in ureteroscopic approach to renal stones clearly led to an increase in popularity of URS in treating staghorn stones in selective cases. These technological innovations include the use of nitinol baskets and holmium:YAG laser and greater delectability and durability of the instrument [1].

In a recent study comparing combined ureteroscopic laser fragmentation of stone and PCNL to multiple-access PCNL in the treatment of struvite staghorn stones, the former was associated with less blood loss and generally good stone clearance rate [33]. Similarly, another study reported no major complications and 78% complete stone clearance of staghorn stones in 9 patients treated with combined URS and lower pole single-tract PCNL [34].

#### ESWL

It is largely known that ESWL is commonly used to treat urinary stones with good results; however, when it comes to treatment of struvite staghorn stones, this not always true. In fact, it has been repeatedly shown that the results of ESWL as monotherapy for staghorn stones are not promising (stone clearance rate ranging from 18% to 67%) [25,35].

Significant risk of complications following ESWL as monotherapy for staghorn stones is reported, including urinary sepsis, stenistrasse and acute urinary obstruction, colicky renal pain and, lastly, perinephric hematoma [1]. In 2006, a retrospective study of 92 renal units with partial staghorn stones treated with ESWL demonstrated the need for more than one session in majority of patients (86%) to achieve an overall stone clearance rate of 60%. This study also reported major ESWL-related complications rate of 13% and the need for unplanned additional procedure in 18.4% of cases [36].

#### Combination ‘sandwich’ therapy

In 1980s, a procedure by which combination of PCNL and ESWL, in certain order, to treat staghorn stones was described. This modality of treatment is still available and it is called sandwich therapy. Sandwich therapy typically involves an initial PCNL reducing the stone load, followed by ESWL to stone fragments that are difficult to access endoscopically. ESWL, in turn, precedes a second PCNL to extract residual stone fragments [37]. Analysis of the outcome of 100 patients who had sandwich therapy for struvite staghorn stones revealed complete stone clearance in almost two thirds of the patients, however with an average hospital stay of 12.2 days and the necessity for blood transfusion in 14% of cases [37].

Although another study reviewing the results of 101 patients who undergone sandwich therapy for staghorn stones showed slightly better stone-free rate (67%), interestingly, these figures are still lower than complete stone clearance rate following PCNL only [38]. For this reason, combination or sandwich therapy has become less popular compared to PCNL monotherapy.

#### Anatrophic nephrolithotomy (AN)

In 1960s, AN was described based on the principle of bloodless plane of incision between anterior and posterior segmental renal vessels. The avascular plane of renal incision was first demonstrated using methylene blue injection after clamping posterior branches of the renal artery [39].

Currently, AN can only be considered as a treatment option for staghorn stones if clearance of staghorn stone is not possible with multiple PCNL procedures with or without ESWL, PCNL and ESWL are proved to be challenging with generally poor results, staghorn stone is excessively large (surface area of >2500 mm^2^) or the pelvicalyceal system is markedly dilated [40].

#### Chemolysis or stone dissolution therapy

Since 1932, attempts at dissolving struvite staghorn stones have been recorded. Few years later, a successful chemolysis of a renal stone was confirmed using permanganate and boric acid [15,41]. Chemolysis was not popular until 1943, when Suby’s solution was developed and later upgraded to Suby’s solution G. The later is composed of sodium carbonate, magnesium oxide, and citric acid [42]. Citric acid breaks down to hydrogen and citrate that binds to calcium and phosphate from the stone with resultant calcium citrate and phosphoric acid, respectively [41]. Hemiacidrin chemolysis via a nephrostomy tube in combination with ESWL for struvite stones in 118 patients achieved complete stone clearance in 60% of cases. Although relatively long hospital stay (mean of 32 days) was necessary in those patients, cheomlysis-related complication rate was noticeably low [43].

Due to the risk of sepsis and electrolyte disturbance, precautions should be undertaken prior to any attempts at using dissolution therapy for staghorn stones. For instance, minimum intrarenal pressure, sterile urine, and regular measurement of serum phosphate and magnesium are necessary before the start of treatment. Also, the administration of prophylactic antibiotics before, during and after dissolution therapy is advocated [42]. Today percutaneous chemolysis is rarely used. Percutaneous irrigation chemolysis may be an option for infection- and uric acid stones.

Stones composed of uric acid, but not sodium or ammonium urate, can be dissolved by oral chemolysis. Prior stone analysis may provide information on stone composition. Urinary pH measurement and X-ray characteristics may provide information on the type of stone.

#### Stone recurrence prevention

Different options are available to reduce the risk of stone recurrence, including dietary advice, antibiotics to sterilize the urine and oral urease inhibitors.

#### Dietary modification

A low phosphate and calcium diet together with estrogen supplement and aluminium gel have been suggested in 1940s to reduce the risk of developing staghorn stones [44]. The role of dietary advice in inhibiting staghorn stone formation is studied in 1968 and reported marked decline in stone recurrence following nephrolithotomy procedure in those who actually follow the dietary regimen [45].

#### Urease inhibitors

Agents that prevent struvite stone formation via supersaturation, also called ‘urease inhibitors’, were first identified in 1960s. Interestingly, till now, only one urease inhibitor is approved for staghorn stone prevention – acetohydroxamic acid (AHA) [42]. Enough evidence is currently available to support the ability of AHA to interrupt struvite stone growth. Nevertheless, serious systemic side effects related to AHA is responsible for high rate of treatment discontinuation (20%) [37,38]. In addition, urease inhibitors toxicity is accentuated by the presence of renal failure and, therefore, AHA should not be used in patients with poor renal function [46].

#### Antibiotic therapy

Although persistent urinary tract infection is a well-known contributor to staghorn stone recurrence and, hence, the importance of the antimicrobial therapy in stone recurrence prevention, a cohort study in 1985 concluded that remnant stone fragments render the ability of antibiotics to eradicate the infection relatively weak [47–49]. The later means that complete stone clearance is more important than antimicrobial therapy in prevention of stone recurrence.

## Conclusions

- Staghorn stones are large and branching stones that fill part or all of the pelvicalyceal system. They are usually unilateral and less common in men. They are linked to urease-producing bacterial infections and, hence, known as struvite infection stones.
- Urinary tract obstruction, long-term urethral catheter, previous urinary diversion, and neurogenic bladder are well-recognized predisposing factors.
- Recent increase in metabolic component of staghorn stones is noticed with calcium phosphate being the most common. However, no clear etiological explanation is available.
- Untreated staghorn stones are detrimental to the kidney and treatment objectives are complete stone removal, successful treatment of the causative bacteria, preserving renal function and prevention of stone recurrence.
- PCNL should be the recommended first-line treatment for staghorn stones. Other surgical and non-surgical treatment options can be considered as a multimodal therapy or separately in certain circumstances.
- Although measures to prevent staghorn stone recurrence are currently available, their clinical utility is under debate, suggesting further research.
